# Differential Induction of Reactive Oxygen Species and Expression of Antioxidant Enzymes in Human Melanocytes Correlate with Melanin Content: Implications on the Response to Solar UV and Melanoma Susceptibility

**DOI:** 10.3390/antiox11061204

**Published:** 2022-06-20

**Authors:** Parth R. Upadhyay, Renny J. Starner, Viki B. Swope, Kazumasa Wakamatsu, Shosuke Ito, Zalfa A. Abdel-Malek

**Affiliations:** 1 Division of Pharmaceutical Sciences, College of Pharmacy, University of Cincinnati, Cincinnati, OH 45267, USA; upadhypr@mail.uc.edu; 2 Department of Dermatology, University of Cincinnati, Cincinnati, OH 45267, USA; starnerj@ucmail.uc.edu (R.J.S.); swopevk@ucmail.uc.edu (V.B.S.); 3 Institute for Melanin Chemistry, Fujita Health University, Toyoake 470-1192, Japan; kazu.chem2518@gmail.com (K.W.); sito@fujita-hu.ac.jp (S.I.)

**Keywords:** human melanocytes, eumelanin, pheomelanin, solar UV, oxidative stress, melanoma risk, reactive oxygen species, antioxidant enzymes, lipid peroxidation

## Abstract

Constitutive pigmentation determines the response to sun exposure and the risk for melanoma, an oxidative stress–driven tumor. Using primary cultures of human melanocytes, we compared the effects of constitutive pigmentation on their antioxidant response to solar UV. The quantitation of eumelanin and pheomelanin showed that the eumelanin content and eumelanin to pheomelanin ratio correlated inversely with the basal levels of reactive oxygen species (ROS). Irradiation with 7 J/cm^2^ solar UV increased ROS generation without compromising melanocyte viability. Among the antioxidant enzymes tested, the basal levels of heme oxygenase-1 (HO-1) and the glutamate cysteine ligase catalytic subunit and modifier subunit (GCLC and GCLM) correlated directly with the eumelanin and total melanin contents. The levels of HO-1 and GCLM decreased at 6 h but increased at 24 h post–solar UV. Consistent with the GCLC and GCLM levels, the basal glutathione (GSH) content was significantly lower in light than in dark melanocytes. The expression of *HMOX1*, *GCLC*, *GCLM*, and *CAT* did not correlate with the melanin content and was reduced 3 h after solar UV irradiation, particularly in lightly pigmented melanocytes. Solar UV increased p53 and lipid peroxidation, which correlated inversely with the eumelanin and total melanin contents. These intrinsic differences between light and dark melanocytes should determine their antioxidant response and melanoma risk.

## 1. Introduction

Exposure to solar ultraviolet radiation (UV) is a major etiological factor, and skin pigmentation is a main determinant of the risk for cutaneous melanoma [1]. It is well–known that the incidence of cutaneous melanoma is much higher in individuals with a light complexion, who burn rather than tan upon sun exposure, than in individuals with a dark skin color, who tan readily [2,3]. According to the American Cancer Society, the overall lifetime risk for cutaneous melanoma is 1 in 38 for Whites, compared to 1 in 1000 in Blacks and 1 in 167 in Hispanics. Human skin pigmentation is determined by the amount of the pigment melanin synthesized by epidermal melanocytes and the transfer of mature melanin containing organelles, melanosomes, to the surrounding keratinocytes [4]. Human melanocytes synthesize two types of melanin: the dark brown eumelanin and the red–yellow pheomelanin [5,6,7]. The eumelanin content correlates directly with skin pigmentation [6,7] and is photoprotective. It is resistant to photodegradation, and eumelanin–rich melanosomes remain intact throughout the epidermal layers, thereby shielding the skin from impinging solar UV rays. Eumelanin is also efficient in quenching reactive species [8,9]. Pheomelanin, on the other hand, is photolabile, a prooxidant, and is potentially oncogenic [8,10]. The differences in the properties of eumelanin versus pheomelanin provide an explanation for the increased risk of cutaneous melanoma in Whites, who have a low eumelanin content and a low eumelanin–to–pheomelanin ratio, and the reduced melanoma risk and sensitivity of individuals with dark skin to solar UV [6].

Solar UV results in the induction of DNA photoproducts and the generation of reactive oxygen species (ROS) and nitrogen species that cause oxidative damage to DNA, proteins, and lipids. The induction of DNA photoproducts correlates inversely with the eumelanin content of cultured human melanocytes and skin [11,12]. However, the effects of the eumelanin and pheomelanin contents on the induction of reactive species and oxidative damage in human melanocytes has not been fully investigated. There is extensive evidence that oxidative stress is a driver of melanomagenesis. Dysplastic nevi, which are precursors for melanoma, have high levels of pheomelanin, ROS, and DNA damage [13,14]. Melanocytes and melanoma tumor cells from melanoma patients are hypersensitive to oxidizing agents, due to an endogenous antioxidant imbalance [15,16], and loss-of-function polymorphisms of glutathione *S*-transferase (GST) antioxidant genes are associated with increased melanoma risk [17]. The activating Braf^V600E^ mutation, which is common in melanoma, is typical of an oxidative stress-induced mutation [18,19]. The generation of ROS and peroxynitrites, mainly in mouse melanocytes that only synthesize pheomelanin, contributes to the delayed induction of cyclobutane pyrimidine dimers, the major form of DNA photoproducts, post-UV exposure, which increases the risk for UV signature mutations that are prevalent in melanoma [20]. Moreover, the synthesis of pheomelanin, in the absence of eumelanin, in recessive yellow mice harboring a loss-of-function mutation in *melanocortin 1 receptor* (*mc1r)* and the V600E mutation in Braf, results in excessive oxidative DNA damage and lipid peroxidation that induce spontaneous melanoma tumor formation [10]. These results demonstrate the genotoxic effects of pheomelanin.

Based on the above findings, this study was conducted to compare the intrinsic properties of lightly pigmented versus dark melanocytes that determine their response to oxidative stress induced by solar UV. The goal is to further elucidate the role of melanin in the antioxidant response of melanocytes, which determines their risk for melanoma. For that, we compared the responses of primary normal human melanocyte cultures derived from donors with different pigmentary phenotypes to oxidative stress induced by solar UV. We quantified the basal eumelanin, pheomelanin, and total melanin contents and compared these cultures for the basal ROS levels and solar UV–induced generation of ROS and the expression of a panel of antioxidant enzymes, namely heme oxygenase-1 (HO-1), glutamate-cysteine ligase modifier (GCLM) and catalytic subunit (GCLC), superoxide dismutase 2 (SOD2), glutathione peroxidase 4 (GPx4), ferritin (FTH1), and catalase. We also compared the levels of glutathione (GSH), the solar UV–induced p53 protein, and the extent of lipid peroxidation in these melanocyte cultures with different melanin contents. The differences we observed between the cultures with low versus high eumelanin and the total melanin content provide an explanation for the increased sensitivity of the former to the oxidative effects of solar UV and their higher risk for melanoma.

## 2. Materials and Methods

### 2.1. Primary Human Melanocyte Cultures

Primary human melanocyte cultures were established from 16 neonatal foreskins from anonymous newborns with different pigmentary phenotypes. Due to the deidentified nature of the foreskins, which are otherwise discarded, the Institutional Review Board at the University of Cincinnati deemed collecting these skin samples exempt from approval. Eight of these cultures were derived from lightly pigmented foreskins, designated as (L), and eight cultures were derived from dark foreskins, designated as (D). The purity of the primary melanocyte cultures was achieved by treating the cultures once or twice a week for 1 to 2 weeks with 100 µg/mL geneticin (G418 sulfate) to remove the fibroblast contamination. The purity of the primary melanocyte cultures was confirmed visually by light microscopy and by immunostaining for the melanocyte marker TRP-1. All cultures were maintained under identical conditions with the melanocyte growth medium consisting of MCDB 153 supplemented with 4% fetal bovine serum, 5 µg/mL insulin, 14 µg/mL bovine pituitary extract, 5 ng/mL phorbol 12-myristate-12-acetate (TPA), 0.75 ng/mL human recombinant basic fibroblast growth factor, and 1% antibiotics/antimycotics, as described previously [21]. All cultures were tested for responsiveness to α–melanocyte–stimulating hormone (α-MSH), the agonist for MC1R, to rule out any increased vulnerability to oxidative stress imposed by the loss of function of MC1R [21,22]. For all experiments, proliferating melanocyte cultures from passage numbers 3–10 were used.

### 2.2. Chemical Analysis of Melanins

Melanocyte samples (2 million) in duplicates were ultrasonicated in 400 H_2_O. Aliquots of 100 µL from each replicate were subjected to alkaline hydrogen peroxide oxidation [23,24] and hydroiodic acid hydrolysis [25]. Eumelanin, benzothiazole-pheomelanin (BZ-pheomelanin), and benzothiazine-pheomelanin (BT-pheomelanin) were calculated by multiplying with conversion factors of 38, 34, and 9 for pyrrole-2,3,5-tricarboxylic acid, thiazole-2,4,5-tricarboxylic acid, and 4-amino-3-hydroxyphenylalanine, respectively [26].

### 2.3. Solar UV Irradiation

Cultured human melanocytes from different donors were plated, and 48 h thereafter, the respective experimental groups were irradiated using a solar simulator (Oriel^®^ Sol-UV 6™; Newport Corporation, Irvine, CA, USA), which emits the entire spectrum of solar UV, with ≥90% in the UVA (320–400 nm wavelength) and 5–10% in the UVB range (280–320 nm wavelength), thereby faithfully simulating real-life sun exposure. For all experiments, unless otherwise specified, 7 J/cm^2^ solar UV was used, which is equivalent to 10 standard erythema doses (SED), and 2 minimum erythemal doses for skin phototype II. The FS-20 lamps (National Biological, Beachwood, OH, USA) that have 75% emissions in the UVB rage and 25% in the UVA range, equipped with a Kodacel filter to shield any UVC rays, were used at doses that were previously reported to induce ROS [22,27]. Melanocytes were irradiated in PBS and then maintained in fresh media after irradiation for the designated time periods.

### 2.4. Viability Assay

To determine the effects of 7 J/cm^2^ solar UV on melanocyte viability and toxicity, melanocytes from 3 L (L1, L5, and L6) and 3 D (D3, D4, and D5) cultures were plated into 24-well plates in triplicates at a density of 50,000 cells/well. After 48 h, cells were irradiated with 7 J/cm^2^ solar UV, as described above, followed by incubation in fresh culture medium. After 48 h post-irradiation, light microscopic images of the control and solar UV-irradiated melanocytes were obtained at 10× magnification. Immediately afterwards, the MTT reagent (Cat. No. M6494; Invitrogen, Waltham, MA, USA) was added directly into the medium at a final concentration of 0.5 mg/mL for 2 h. The medium was then removed, and 200 µL isopropanol was added to each well to solubilize formazan for 10 min on a shaker. Duplicate samples from each well were then aliquoted into 96-well plates, and the absorbance was read using a plate reader at the 570 nm wavelength.

### 2.5. Measurement of ROS

To determine the basal and solar UV-induced ROS levels in 8 L and 8 D melanocyte cultures, the cells were plated at a density of 4 × 10^5^ cells/60 mm dish, with triplicate dishes/group. After 48 h, the cells were washed twice with PBS and loaded with 5 μM of a CM-H_2_DCFDA florescent probe (Cat. No. C6827; Invitrogen, Waltham, MA, USA) for 30 min at 37 °C. The fluorescent probe was then removed, and PBS added, and melanocytes were either unirradiated (control) or irradiated with a solar simulator or FS-20 lamps at the indicated doses and incubated at 37 °C for 1 h in fresh media. Melanocytes from each dish were harvested, washed with PBS, and immediately analyzed as live cells by flow cytometry (10,000 events per sample) using FACS Calibur (Becton Dickinson, Franklin Lakes, NJ, USA), with the median fluorescence intensity detected on the FL-1 filter (excitation: 488 nm, emission: 525 nm) to quantify the cellular levels of ROS.

### 2.6. Quantitation of Reduced GSH

To quantify the basal-reduced GSH concentration in 5 L (L1, L2, L4, L5, and L6) and 4 D (D2, D3, D4, and D5) melanocyte cultures, 1 × 10^6^ cells were plated in 100 mm^2^ dishes. After 48 h, cell lysates were prepared using mammalian cell lysis buffer (Cat. No. ab179835; Abcam, Boston, MA, USA). The cell lysates were then deproteinized and neutralized using a deproteinizing sample preparation kit (Cat. No. ab204708; Abcam, Boston, MA, USA). A GSH/GSSG ratio detection kit (Cat. No. ab205811; Abcam, Boston, MA, USA) was used to determine the levels of reduced GSH concentration in each sample, as determined by a linear regression analysis of the GSH standard curve.

### 2.7. Western Blot Analysis

A Western blot analysis was carried out to compare the protein levels of HO-1, GCLC, GCLM, SOD2, GPX4, FTH1, catalase, and p53 in 6 L and 5 D melanocyte cultures. Melanocytes were plated in 100 mm dishes at a density of 1.25 × 10^6^ cells per dish. Forty-eight hours thereafter, the growth medium was removed and replaced by PBS, and the melanocytes were either irradiated with 0 (control) or 7 J/cm^2^ solar UV. PBS was then removed, and melanocytes maintained in fresh growth medium at 37 °C. Total proteins were extracted at 6 and 24 h post-irradiation using RIPA buffer containing a cocktail of protease and phosphatase inhibitors. Primary antibodies specific for the above-listed proteins that were used for immunoblotting are included in Supplementary Table S1. Molecular weight markers (Bio-Rad Precision Plus Protein Western C Standards, Cat. No. 1610399; Bio-Rad, Hercules, CA, USA) were run to ensure the accuracy of the detected bands. A densitometry analysis was carried out using Image-lab software (Bio-Rad, Hercules, CA, USA), normalizing each band to its respective GAPDH or actin loading control.

### 2.8. Real-Time Polymerase Chain Reaction (RT PCR)

RT-PCR was performed to determine the mRNA expression levels in 2 L and 3 D primary human melanocyte cultures. Cells were plated in 100 mm dishes at a density of 1.25 × 10^6^ cells per dish in melanocyte growth medium. Forty-eight hours thereafter, melanocytes were irradiated with 0 (control) or 7 J/cm^2^ solar UV, then received fresh medium and were incubated at 37 °C. After 3 h, the cells were harvested and total RNA was extracted using the Qiagen RNeasy Mini Kit (Cat No. 70104, Qiagen, Germantown, ML, USA), according to the manufacturer’s protocols. Melanin was removed from extracted RNA by standard phenol-chloroform clean-up, followed by the precipitation of RNA by sodium acetate and ethanol. The purity and quantitative analysis of RNA were determined using NanoDrop 2000 (Thermo Fisher Scientific, Waltham, MA, USA). One microgram of RNA from each sample was then converted into cDNA using the SuperScript^TM^ VILO^TM^ cDNA synthesis kit (Cat No. 11754050; Invitrogen, Waltham, MA, USA) following the manufacturer’s instructions. Real-time PCR was performed with cDNA using the PowerSYBR Green PCR Master Mix (Cat No. 4367659; Applied Biosystems, Woolston, UK). RT-qPCR Primers were purchased from Qiagen (Cat No. 330001; Qiagen, Germantown, ML, USA) for *HMOX1* (GeneGlobe ID: PPH00161F-200), *GCLM* (GeneGlobe ID: PPH02099A-200), *GCLC* (GeneGlobe ID: PPH00482A-200), *CAT* (GeneGlobe ID: PPH00420B-200), and *GAPDH* (GeneGlobe ID: PPH00150F-200).

### 2.9. Immunofluorescence Staining for Lipid Peroxidation

Basal and solar UV-induced lipid damage in 3 L (L1, L5, and L6), and 3 D (D3, D4, and D5) melanocyte cultures was determined using the Click-IT lipid peroxidation imaging kit (Cat. No. C10446; Invitrogen, Waltham, MA, USA) according to the manufacturer’s instructions. Briefly, melanocytes were plated on serum-coated glass coverslips at a density of 4 × 10^4^ cells/cover slip. After 48 h, the culture medium was removed and replaced with PBS, and the melanocytes were irradiated with 0 or 7 J/cm^2^ solar UV. After 1 h, the melanocytes were fixed with 4% paraformaldehyde, permeabilized using 0.1% Triton-X-100, and blocked with 1% bovine serum albumin (BSA) for 30 min at room temperature. A Click-IT reaction mixture, consisting of reaction buffer, CuSO_4_, Alexa Fluor 488, and additive buffer, was added and incubated for 30 min at room temperature. Melanocytes were then washed twice with 1% BSA and twice with PBS. Cellular nuclei were stained using 2 µg/mL Hoechst 33342. Coverslips were then mounted on glass slides, visualized by fluorescence microscopy, and images were acquired using a Nikon Eclipse 90 microscope (Tokyo, Japan). Image analysis was performed using Nikon NIS-Elements AR software.

### 2.10. Statistical Analysis

Data were statistically analyzed using ANOVA, followed by Tukey’s multiple comparison test, or an unpaired *t*-test using GraphPad Prism 6 (GraphPad Software, San Diego, CA, USA).

## 3. Results

### 3.1. Comparison of Eumelanin and Pheomelanin Contents in Melanocyte Cultures with Different Pigmentation

**Figure 1:**
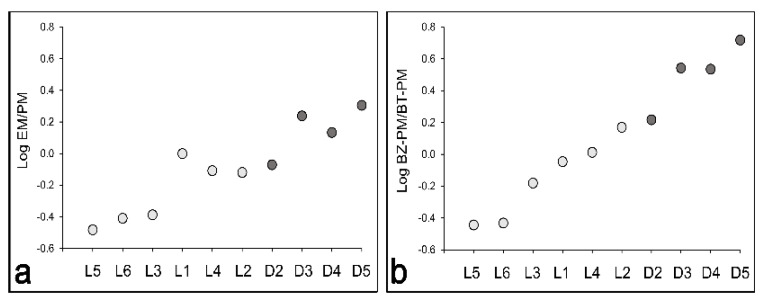
Comparison of the eumelanin (EM)-to-pheomelanin (PM) ratio in (**a**) and the ratio of benzothiazole-pheomelanin (BZ-PM) to benzothiazine-pheomelanin (BT-PM) in (**b**) in L versus D melanocyte cultures.

The contents of eumelanin, as well as pheomelanin, in the form of benzothiazine (BT)- and benzothiazole (BZ)-pheomelanin were quantified in six L and four D melanocyte cultures (Table 1 and Figure 1). The eumelanin content was consistently higher in the visually D cultures than in their L counterparts (Table 1). These results are consistent with our previously published data that the eumelanin content correlates directly with the visual pigmentation [7]. The total pheomelanin content, particularly BZ-pheomelanin, was also higher in D than in L melanocytes (Table 1). Benzothiazine moieties are unstable, and upon degradation, BZ moieties, which are more stable and have a lower oxidation potential, are formed [28,29]. The ratio of eumelanin to pheomelanin was lower in the L cultures (ranging between 0.33 and 0.78 in five of the six cultures) than in the D cultures (where it ranged between 0.85 and 2.02) (Figure 1a). Interestingly, one lightly pigmented culture, L1, had the same low eumelanin and pheomelanin contents, which resulted in a eumelanin-to-pheomelanin ratio of 1. The lowest eumelanin-to-pheomelanin ratio among the D cultures was that of an intermediately pigmented culture, D2, which had a higher total pheomelanin than eumelanin content, yet still had a higher eumelanin content than any of the L cultures (Table 1 and Figure 1a). The quantitation of BZ- and BT-pheomelanin revealed that the former represents the predominant pheomelanin in the D but not in the L melanocyte cultures, which consistently had a lower BZ- to BT-pheomelanin ratio than the D melanocyte cultures (Table 1 and Figure 1b).

### 3.2. Comparison of Basal and Solar UV-Induced ROS in L versus D Melanocyte Cultures

**Figure 2:**
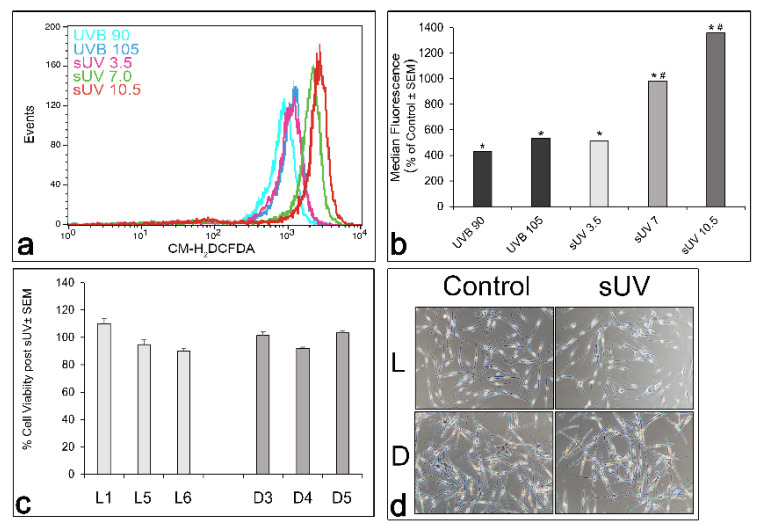
Dose-dependent generation of ROS in response to UVB versus solar UV (sUV) in lightly pigmented melanocytes, and the impact of solar UV on the viability of L and D melanocytes. In (**a**), the histogram represents comparison of the dose-dependent increase in ROS by 90 and 105 mJ/cm^2^ UVB and 3.5, 7, and 10 J/cm^2^ solar compared to the control, as determined following CM-H_2_DCFDA loading, and a flow cytometry analysis 1 h post-UV irradiation, as described in Materials and Methods. (**b**) The quantitation of the data in (**a**), expressed as the mean ± SEM of triplicate samples/group. * = Statistically significantly different from the respective control group (*p* < 0.001); # = statistically significantly different from UVB groups and the other solar UV-irradiated groups (*p* < 0.001). (**c**) The effect of 7 J/cm^2^ solar UV on the viability of L versus D melanocytes, as measured by the MTT assay 48 h post-irradiation. Each bar represents the mean cell viability of triplicate samples ± SEM. (**d**) Light microscopic view (10× magnification) of the control and solar UV-irradiated L and D melanocytes 48 h post-irradiation with 7 J/cm^2^ solar UV.

Irradiation with solar UV resulted in dose-dependent generation of ROS, and solar UV, which is predominantly UVA, was more efficient in inducing ROS than UVB (Figure 2a,b). A dose of 7 J/cm^2^ solar UV caused a significantly greater increase in the generation of ROS than the 90 or even 105 mJ/cm^2^ UVB that we had used in previously published studies (Figure 2b) [22,30]. The latter dose of UVB induced substantial hydrogen peroxide and 8-oxo-deoxyguanosine, the major form of oxidative DNA damage, in human melanocytes [22]. The dose of 7 J/cm^2^ solar UV did not significantly compromise the melanocyte viability in any of the L or D cultures, as determined by the MTT assay 48 h post-irradiation (Figure 2c), and changes in the cell morphology, evidenced by increased dendrite formation and dendrite length, were only noted in the D melanocytes (Figure 2d).

**Figure 3:**
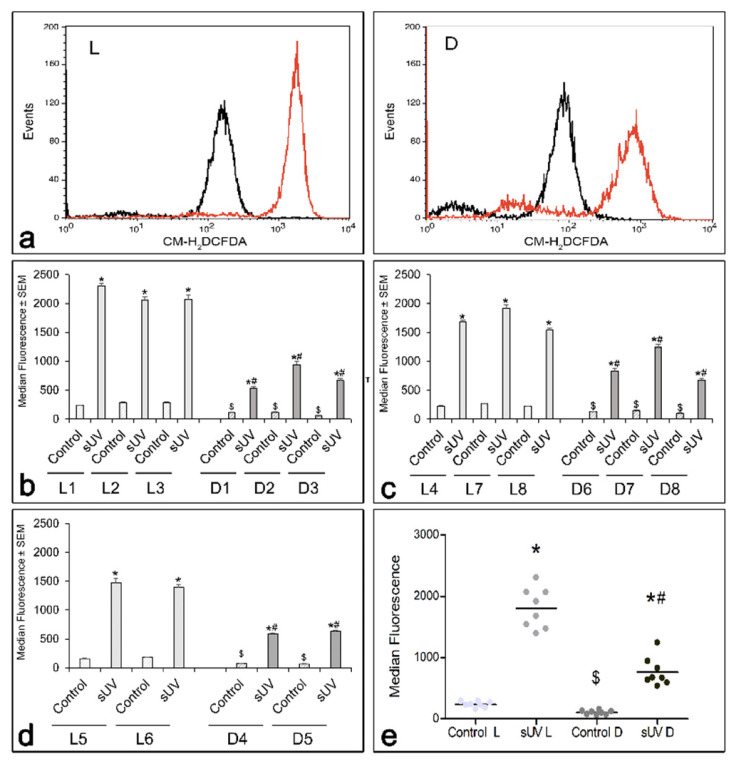
Comparison of basal and 7 J/cm^2^ solar UV-induced ROS in 8 L versus 8 D melanocyte cultures. (**a**) Includes representative histograms comparing basal (in black) and solar UV-induced (in red) ROS in L and D melanocytes. (**b**–**d**) Represent the results of 3 independent experiments, as determined after loading with CM-H_2_DCFDA and a flow cytometry analysis, as described in Materials and Methods. (**e**) Dot plot representing the cumulative data in (**b**–**d**). * = Significantly different from their respective control groups (*p* < 0.0001). $ = Significantly different from the control L groups (*p* < 0.0001). # = Significantly different from all irradiated L groups and *p* < 0.0001.

Comparison of a total of eight L to eight D melanocyte cultures revealed that the basal levels of ROS in the control unirradiated cultures correlated inversely with the eumelanin and total melanin contents and were consistently significantly higher in the L cultures (Figure 3b–e). All cultures, regardless of the melanin content, responded to the same dose of solar UV with a statistically significant increase in ROS. In general, the percent increase in ROS above the control did not correlate with the melanin content (Supplementary Figure S1). Although the D melanocytes showed a substantial increase in solar UV-induced ROS, the absolute ROS levels were significantly lower than in the L melanocytes, with a lower total melanin and eumelanin contents, which are expected to result in greater oxidative stress (Figure 3b–e).

### 3.3. Basal and Solar-UV Induced Levels of Antioxidant Enzymes and Basal GSH Content in L versus D Melanocyte Cultures

**Figure 4:**
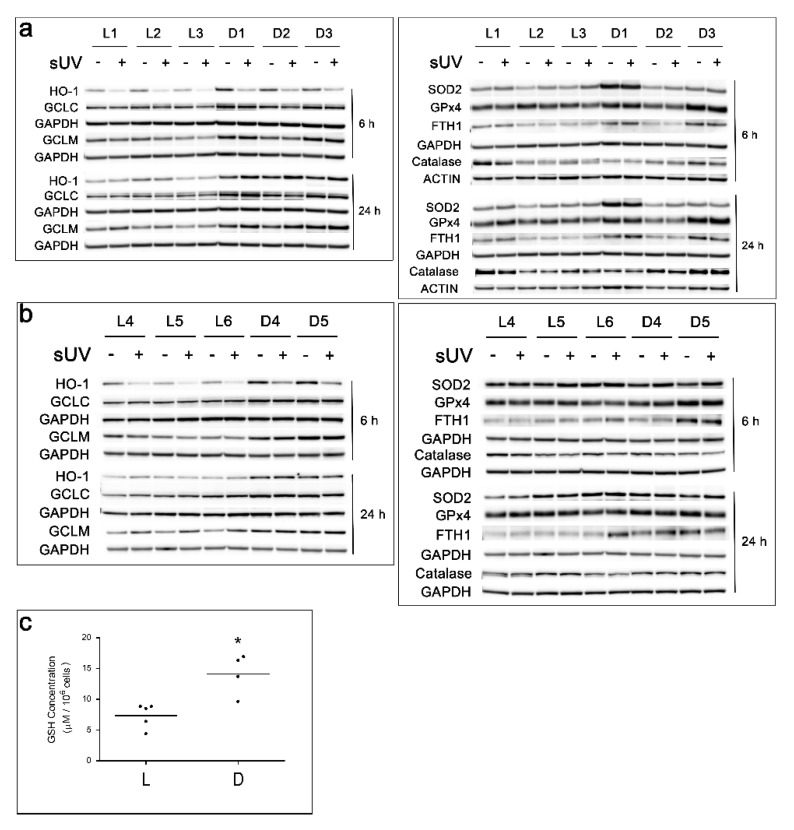
Comparison of the basal and solar UV-induced levels of the antioxidant enzymes HO-1, GCLC, GCLM, SOD2, GPx4, FTH1, and catalase in 6 L and 5 D melanocyte cultures, as determined 6 and 24 h post-irradiation with 7 J/cm^2^ solar UV by Western blot analysis of the total proteins and quantitation of the basal levels of reduced GSH in 5 L and 5 D cultures. (**a**,**b**) The results of 2 independent experiments. GAPDH and actin were used as the loading controls. (**c**) Comparison of basal-reduced GSH levels in L1, L2, L4, L5, and L6 versus D2, D3, D4, and D5. * = Mean GSH content in D cultures is statistically different from that in L cultures (*p* < 0.01).

We compared the protein levels of the antioxidant enzymes HO-1, GCLC, GCLM, SOD2, GPx4, FTH1, and catalase in the control and solar UV-irradiated melanocytes at 6 and 24 h post-irradiation by Western blotting, followed by densitometry and statistical analysis (Figure 4a,b and Supplementary Figures S2 and S3). Heme oxygenase-1 catalyzes the cleavage of heme groups, leading to the generation of the strong antioxidant biliverdin, carbon monoxide, and the release of ferrous ions [31]. This enzyme seems to be of particular significance for melanocytes, where its gene expression is greater and more highly inducible by oxidative stress than in keratinocytes [32,33]. GCLC and GCLM are the two subunits of GCL that catalyze the first and rate-limiting step for the synthesis of GSH, the most predominant intracellular thiol antioxidant, namely the conversion of glutamyl and cysteine to γ-glutamyl cysteine [34]. SOD2 dismutates superoxide into the less reactive hydrogen peroxide, which is then converted by catalase to water and oxygen [35]. GPx4 reduces hydrogen peroxide, and primarily lipid peroxides, and plays a central role in protecting cells from lipid peroxidation and ferroptosis [36]. Ferritin sequesters iron, which otherwise can catalyze ROS generation via the Fenton reaction [37].

In two independent experiments, a total of six L and five D melanocyte cultures were compared for the expression of the above antioxidant enzymes. Melanocytes with a low melanin content expressed lower basal levels of HO-1, GCLC, and GCLM than melanocytes with a high melanin content (Figure 4a,b and Supplementary Figures S2 and S3). The observed differences in the basal GCLC and GCLM protein levels correlated with the basal reduced GSH content in L versus D melanocytes, with L1, L2, L4, L5, and L6 cultures having significantly lower basal levels of GSH than D3, D4, and D5 cultures (Figure 4c). The intermediately pigmented D2 culture had a GSH content comparable to that of the L1, L5, and L6 counterparts and lower than that of the remaining three D cultures.

Irradiation with solar UV resulted in an initial reduction within 6 h in the levels of HO-1 in all cultures tested and a slight reduction in GCLM, particularly in lightly pigmented cultures (Figure 4a,b and Supplementary Figure S2). By 24 h post-solar UV, the levels of HO-1 increased, almost reaching the basal levels, and the levels of GCLM increased above the basal levels in all cultures. Irradiation with solar UV had less effect on GCLC, as compared to GCLM and HO-1. There was no correlation between the melanin content and the basal levels of SOD2, GPx4, FTH1, or catalase (Figure 4a,b and Supplementary Figure S2). Irradiation with solar UV increased the levels of SOD2 in all cultures at 6 h post-irradiation, and the increase remained evident in some of the cultures at 24 h. However, irradiation with solar UV did not alter the level of GPx4 and had inconsistent effects on catalase and FTH1 at both time points.

**Figure 5:**
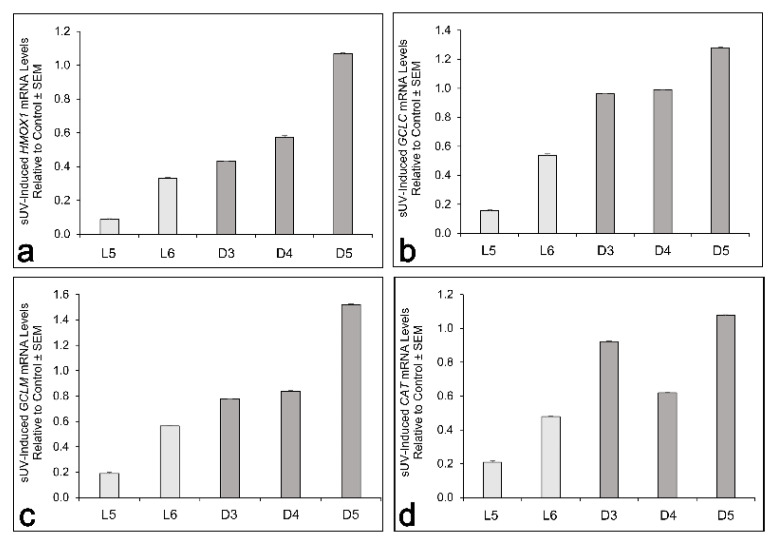
Early effect of solar UV on the mRNA levels of *HMOX1* (**a**), *GCLC* (**b**), *GCLM* (**c**), and *CAT* (**d**), as determined by RT PCR 3 h post-irradiation in 2 L and 3 D melanocyte cultures. Each bar represents the mean mRNA value of triplicate samples/group relative to the control ± SEM.

Using two L and three D melanocyte cultures, we compared by RT PCR the gene expression of *HMOX1*, which codes for HO-1, *GCLC*, and *GCLM*, the protein levels of which were higher in D than in L melanocytes, and *CAT*, the gene that codes for catalase, the protein level of which did not correlate with the total melanin and eumelanin contents (Figure 4a,b and Supplementary Figures S2 and S5). There was no apparent correlation of the mRNA levels of any of these genes with the melanin content. However, 3 h post-solar UV, the reduction in the mRNA levels of all four genes was evident in the two L cultures and was more pronounced than in the three D cultures. The two dark cultures, D3 and D4, showed a reduction in the *HMOX1* and *GCLM* mRNA levels (Figure 5a,c) but not in the mRNA levels of *GCLC* (Figure 5b). Of the three dark cultures, only D4 showed a reduction in *CAT* (Figure 5d). The dark melanocyte culture, D5, which had the highest eumelanin-to-pheomelanin ratio (Figure 1b), showed no reduction in the mRNA levels of any of the four genes in response to irradiation with 7 J/cm^2^ solar UV.

### 3.4. Solar UV-Induced p53 Levels and Lipid Peroxidation in Light versus Dark Melanocyte Cultures

**Figure 6:**
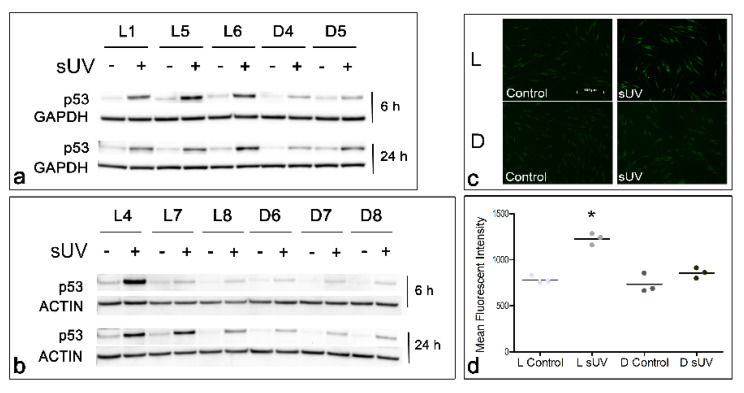
Comparison of the solar UV-induced increase in p53 and lipid peroxidation in L versus D melanocyte cultures. (**a**,**b**) Represent Western blot analysis of p53 in a total of 6 L and 5 D cultures in two independent experiments after 6 and 24 h post-solar UV irradiation. (**c**) Depicts representative control and solar UV-irradiated L and D melanocytes that were stained for lipid peroxidation 1 h post−solar UV. (**d**) Represents a quantitative analysis of the mean fluorescence of the control and solar UV-irradiated melanocytes (a total of 3 L and 3 D cultures) that were stained for peroxidized lipids. L1, L5, L6, D3, and D4, but not D5, responded to solar UV with a statistically significant increase in lipid peroxidation (*p* < 0.001). * = Statistically different from all other groups (*p* < 0.001).

It is established that the transcription factor p53 is increased in response to DNA damage and is the master regulator of the DNA damage response, including cell cycle arrest, DNA repair, and apoptosis, as well as the antioxidant response [38,39,40,41]. Increased ROS generation is known to stabilize and transactivate p53 via activation of the DNA damage sensor ATM [42]. We found that solar UV increased the levels of p53 protein in a time-dependent manner in the L and D cultures, as shown in two independent experiments in which a total of six L and five D cultures were tested (Figure 6a,b and Supplementary Figures S4 and S5). Consistently, L melanocytes showed a greater increase in p53 as compared to D melanocytes. Immunofluorescence staining for lipid peroxidation showed a statistically significant increase in the mean fluorescence in L1, L5, and L6, and in D3 and D4, in response to solar UV, indicating an increased lipid peroxidation (Figure 6c,d). Importantly, all three L cultures had a statistically greater increase in the mean fluorescence staining than their three D counterparts, suggesting that these former cultures had greater lipid peroxidation. One of the three D cultures, D5, with the highest eumelanin-to-pheomelanin ratio (Figure 1a), showed no significant increase in the mean fluorescence staining in response to solar UV, indicating no significant generation of lipid peroxidation.

## 4. Discussion

It has been established that acute exposure to solar UV is an underlying cause of melanomagenesis [1] and that the epidermal melanin content determines the response of melanocytes to solar UV and the risk for melanoma [11,43,44]. Cutaneous melanoma tumors contain somatic mutations in the genes that are associated with the DNA damage response, particularly in repair genes, in addition to an abundance of C > T transitions [45,46]. Given the increasing evidence for the role of oxidative stress in melanomagenesis and the role of melanin in determining the melanoma risk, we conducted this study to compare the antioxidant capacities of human melanocytes derived from different pigmentary phenotypes and their response to the oxidative effects of solar UV. We tested a total of 16 primary normal human melanocyte cultures, 8 derived from lightly pigmented skin and 8 derived from dark skin.

There were several unique aspects in this study. While most previous studies on the oxidative effects of melanin relied on mouse models, where only eumelanin or pheomelanin was synthesized by follicular melanocytes, or on purified or synthetic eumelanin or pheomelanin, we used primary cultures of human melanocytes that synthesize both eumelanin and pheomelanin, as is the case in vivo, to elucidate the combined impact of these types of melanin on the oxidative effects of solar UV. Unlike many other studies that have used different UV spectra (UVA or UVB), we used a solar simulator that captures the entire spectrum of solar UV, thereby simulating real-life sun exposure. We quantified eumelanin and the two forms of pheomelanin, BT- and BZ-pheomelanin, using a HPLC analysis to better characterize the pigmentation of each culture rather than designate them based on the Fitzpatrick phototype of the donor, which can be subjective, or on the use of Mexameter that measures the skin color.

We characterized the pigmentation of six L and four D cultures by quantifying their eumelanin, BT-pheomelanin, BZ-pheomelanin, and total melanin (Table 1 and Figure 1). The eumelanin and total melanin contents consistently correlated with the visually based designation of these cultures, as we have previously reported (Table 1) [7]. Lightly pigmented melanocyte cultures contained less eumelanin and BT- and BZ-pheomelanin and, therefore, less total melanin than their dark counterparts, consistent with the previously published results [26,47,48]. The total pheomelanin content, particularly BZ-pheomelanin, and the ratio of BZ- to BT-pheomelanin were markedly higher in the D than in the L melanocyte cultures (Table 1 and Figure 1b). It is established that BT-pheomelanin is produced primarily by the oxidation of 5-*S*-cysteinyl dopa and converted to BZ-pheomelanin during photolysis or upon exposure to UVA or heat [28,29,49,50]. The enhancement of BZ formation is thought to modify the pigment chromophore, further reducing its photoprotective ability, by a mechanism that still needs to be determined [51].

It has long been known that eumelanin is photoprotective against UV due to its resistance to photodegradation and the persistence of eumelanin containing melanosomes throughout the epidermis of dark human skin, which reduces the penetration of solar UV rays through the skin layers [4,52]. Melanosomes in dark skin form supranuclear caps in epidermal cells, thereby protecting DNA from the damaging effects of impinging UV rays [53]. Moreover, eumelanin is efficient in quenching the reactive radicals, and the diffusible eumelanin metabolite DHICA has been shown to have antioxidant effects [9,54]. In lightly pigmented skin, the predominant pheomelanin-containing melanosomes are degraded and only “melanin dust” is detected in the epidermis [4]. Pheomelanin is susceptible to photodegradation and is phototoxic due to its ability to convert molecular oxygen to superoxide when exposed to UV [55,56]. Following UVA irradiation, pheomelanin can act as a photosensitizing agent, inducing charge modifications of native catalase, by a mechanism involving singlet oxygen [57]. The increasing epidemiological evidence linking the light skin and red hair phenotype to the expression of loss-of-function variants of *MC1R*, the principal regulator of eumelanin synthesis, and to an increased melanoma risk sparked further interest in investigating the roles of pheomelanin and eumelanin in determining the response of melanocytes to UV and the risk of melanoma [58,59,60]. A study on recessive yellow mice harboring a loss-of-function mutation in *mc1r* and the activating Braf^V600E^ mutation found that pheomelanin, exclusively synthesized by melanocytes in this model [61], generated extensive reactive species, oxidative DNA damage, and lipid peroxidation and resulted in spontaneous melanoma tumor formation [10]. These results led to the conclusion that pheomelanin is a strong prooxidant and, on its own, is oncogenic in the absence of any exogenous carcinogen [10]. Syngeneic albino mice expressing the same mutations in *mc1r* and Braf did not have increased oxidative stress, nor did they develop melanoma tumors, suggesting that the presence of pheomelanin is carcinogenic and more detrimental to melanocytes than the absence of melanin. In addition, pheomelanin can deplete cysteine-based antioxidants, such as GSH, thereby contributing further to the vulnerability of melanocytes to oxidative damage and melanomagenesis [62]. This is supported by studies showing that purified pheomelanin from red hair or synthetic pheomelanin directly accelerates the auto-oxidation of GSH and NADPH [63,64]. However, human melanocytes synthesize both eumelanin and pheomelanin, so the combined effects of both melanin must be considered, as they will determine the extent of photoprotection and the oxidative status conferred by the total melanin.

A comparison of the intrinsic ROS levels in L versus D human melanocytes showed that the basal levels of ROS correlated inversely with the total melanin and eumelanin contents (Figure 3). Since the activity of MC1R is an important regulator of eumelanin synthesis and a determinant of the DNA damage response to UV and the risk of melanoma [30,65,66,67], the melanocyte cultures used in this study were all tested and found to respond avidly to α-MSH, indicating that they express functional MC1R. Our results are consistent with those of a previous in vivo study comparing skin phototypes IV and V to skin type II using electron paramagnetic resonance spectrometry, which found that, in response to solar UV, the former had a markedly less generation of free radicals in the skin than the latter [68]. Although we found that irradiation with solar UV markedly increased ROS generation in all melanocyte cultures regardless of their pigmentation, the absolute levels of ROS were by far higher in the L cultures than in their D counterparts, making them more vulnerable to oxidative stress (Figure 3 and Supplementary Figure S1). These results suggest that a high eumelanin content in D melanocytes reduces ROS generation and/or neutralizes ROS and overrides the prooxidant effect of pheomelanin. Conversely, a low eumelanin content in L melanocytes is permissive for the prooxidant effect of pheomelanin.

Among the panel of antioxidant enzymes tested, the levels of HO-1, GCLC, and GCLM were most remarkably different between the L and D melanocyte cultures (Figure 4a,b and Supplementary Figures S2 and S3). Our results showed that L melanocytes had lower basal levels of HO-1, GCLC, and GCLM than D melanocytes. Heme oxygenase-1 seems to be of particular significance for melanocytes, since its gene expression is greater than in keratinocytes, and is highly inducible by oxidative stress [32,33]. The protein levels of GCLM were modulated 24 post-solar UV exposure to a greater extent than GCLC (Figure 4a,b and Supplementary Figure S2). This is not surprising, since GCLM is the essential regulator of the activity of GCL and, therefore, GSH synthesis. Although all cultures responded to irradiation with solar UV with an initial reduction at 6 h post-irradiation, followed by an increase in the HO-1 and GCLM levels, the levels of these enzymes remained much higher in the D cultures, which is expected to reduce their sensitivity to the oxidative effects of solar UV (Figure 4a,b and Supplementary Figures S2 and S3). Consistent with the differences in the GCLC and GCLM levels in L versus D melanocytes, the basal levels of reduced GSH were significantly higher in D than in L melanocytes (Figure 4c). These findings suggest that melanocytes with a high eumelanin content have a more efficient antioxidant response to solar UV than their counterparts with a low eumelanin content.

We did not detect consistent differences in the basal levels of SOD2, GPX4, ferritin, and catalase among the L versus the D cultures (Figure 4a,b and Supplementary Figure S2). Our results differ from those of Maresca et al., who reported a direct correlation between the melanin content on the one hand and catalase expression and activity on the other [69]. In future experiments, we will compare the activities of SOD2 and catalase in melanocytes with different melanin contents and how they might be modulated by solar UV.

RT-PCR data showed the variability of the mRNA levels of *HMOX1*, which codes for HO-1, *GCLC*, *GCLM*, and *CAT*, which codes for catalase, among the melanocyte cultures tested, with no correlation with the melanin content. These results suggest that the differences in the basal levels of these enzymes in L versus D melanocytes, as detected by Western blotting in Figure 4, are not due to differences in gene transcription among the cultures. However, early reduction in the mRNA levels of all four enzymes following solar UV exposure was evident in all cultures, yet was more pronounced in L than in D melanocytes (Figure 5) and led to the initial reduction in the protein levels of HO-1 and GCLM (Figure 4a,b and Supplementary Figure S2). These experiments need to be expanded to determine the possible delayed transcriptional effects of solar UV (e.g., 24 h post-irradiation).

It is established that transcription factor p53 is the master regulator of the DNA damage response and is stabilized and transactivated in response to DNA damage [38,39,40,41]. Increased ROS generation, as observed following solar UV exposure in melanocytes (Figure 3), results in p53 stabilization and transactivation [42]. We previously reported on the significance of p53 in the antioxidant response of human melanocytes [27]. An important finding in this study is that L melanocytes expressed markedly higher levels of p53 in response to solar irradiation than their D counterparts (Figure 6a,b and Supplementary Figures S4 and S5). The accumulation of p53 was evident 6 h post-irradiation, particularly in L melanocytes, and significantly increased further at 24 h post-solar UV (Figure 6a,b and Supplementary Figures S4 and S5). We previously observed similar differences in the p53 levels in L versus D human melanocytes after irradiation with UVB, which correlated directly with the induction of DNA photoproducts [12,70]. Here, the p53 levels correlated with the ROS levels and lipid peroxidation in L versus D melanocytes. We attribute the higher levels of p53 in L melanocytes to more UV-induced oxidative damage and total DNA damage than in their D counterparts.

Another important finding is that L melanocytes encountered significantly more lipid peroxidation than D melanocytes in response to irradiation with solar UV (Figure 6c,d). Interestingly, the extent of lipid peroxidation correlated directly with the solar UV-induced p53, the levels of ROS, and inversely with the levels of HO-1 and GCLM (Figure 3b–e, Figure 4a,b, Supplementary Figures S2 and S3, Figure 6a,b, and Supplementary Figures S4 and S5). It is known that p53 modulates ferroptosis, an iron-dependent, nonapoptotic form of cell death induced by lipid peroxidation in response to high levels of ROS [71]. Increased lipid peroxidation in L melanocytes suggests that low levels of eumelanin allow for the prooxidant effects of pheomelanin, which increase the oxidative damage and lipid peroxidation, consistent with the findings by Mitra et al. [10]. On the other hand, reduced lipid peroxidation in D, relative to L melanocytes suggests that the high eumelanin and total melanin contents reduce oxidative stress and the resulting oxidative damage in melanocytes.

## 5. Conclusions

**Figure 7:**
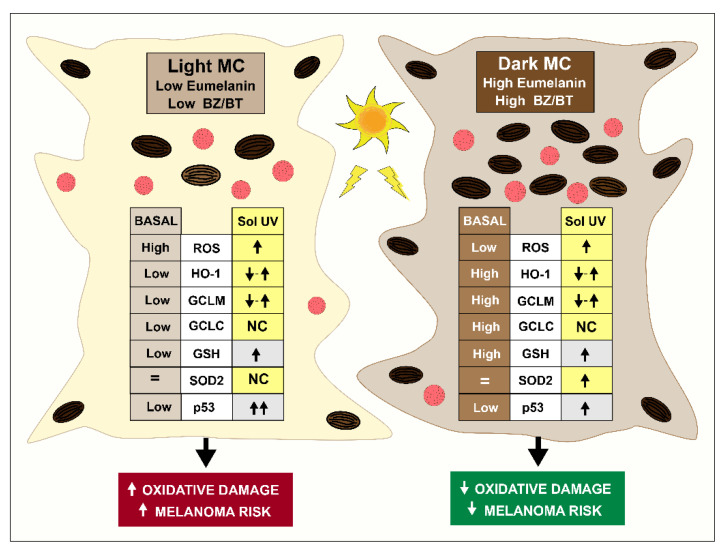
Summary of the observed differences between light and dark melanocytes (MC) that account for the increased oxidative damage and melanoma risk in individuals with light skin. Light melanocytes have higher basal and solar UV-induced levels of ROS and express lower basal levels of the antioxidant enzymes HO-1, GCLM, and GCLC than dark melanocytes. Due to the differences in the GCLM and GCLC levels, the GSH levels are lower in light than in dark melanocytes. In response to solar UV, the levels of HO-1 and GCLM are initially reduced at 6 h but then rebound at 24 h post-irradiation. Following the solar UV exposure, no change in the levels of GCLC was observed following solar UV exposure, and the levels of SOD2 were only increased in dark melanocytes. The solar UV-induced increase in p53 and lipid peroxidation, indicative of oxidative damage, were markedly greater in light, as compared to dark, melanocytes. ↑ = increase; ↓ =decrease; MC, Melanocytes.

There is overwhelming evidence that oxidative stress plays a central role in the different stages of melanomagenesis [72,73]. High levels of ROS contribute to the progression of melanoma from the radial growth to vertical growth phase, and the extent of oxidative DNA damage and lipid peroxidation increases sequentially with the stage of melanoma. In this study, we characterized multiple important intrinsic differences between normal human melanocytes with different melanin contents (summarized in Figure 7), which can account significantly for the differences in their response to solar UV-induced oxidative stress and the risk of melanomagenesis. Our results provide experimental evidence that the melanin content not only determines the extent of DNA photoproducts induced by solar UV [11,12] but, also, the generation of ROS, expression of antioxidant enzymes, synthesis of GSH, activation of p53, and induction of lipid peroxidation, which can affect the genomic stability of melanocytes and increase the risk of melanoma.

The use of antioxidants, particularly mitochondrial-specific antioxidants to restore ROS homeostasis, proved to be efficacious for the treatment of ROS-associated diseases, such as Alzheimer’s and dry eye disease [74,75]. Our data hereby presented suggest that antioxidants might be efficacious in melanoma prevention, as they can prevent the solar UV-induced malignant transformation of normal melanocytes. Paradoxically, the use of the antioxidants *N*-acetyl cysteine and Trolox was shown to enhance melanoma metastasis [76], thereby contributing to melanoma progression. We emphasize that, in this study, we compared the responses to the oxidative effects of solar UV of normal human melanocytes (not premalignant nevi or melanoma cells) that differ in their pigmentation.

## Figures and Tables

**Table 1:** Quantitative analysis of basal Eumelanin (EM), BZ-pheomelanin (BZ-PM), BT-pheomelanin (BT-PM), and total melanin in light (L) versus dark (D) melanocyte cultures. Each data point is the mean of duplicate samples.

Melanin Contents				
Cell Strain	EM (µg/10^6^ cells)	BZ-PM (µg/10^6^ cells)	BT-PM (µg/10^6^ cells)	Total Melanin (µg/10^6^ cells)
L1	1.18	0.56	0.62	2.36
L2	1.83	1.44	0.97	4.24
L3	1.95	1.88	2.83	6.66
L4	4.12	2.70	2.61	9.44
L5	1.09	0.80	2.45	4.35
L6	0.61	0.42	1.13	2.16
D2	18.96	13.94	8.42	41.33
D3	40.15	20.38	5.85	66.38
D4	38.95	22.24	6.48	67.66
D5	30.08	12.53	2.39	45.00

## Data Availability

All of the data is contained within the article and the supplementary materials.

## Supplementary Materials

The following supporting information can be downloaded at https://www.mdpi.com/article/10.3390/antiox11061204/s1: Figure S1: Solar UV-induced increase in ROS. Figure S2: Densitometry analysis of the Western blot data presented in Figure 4. Figure S3: Statistical analysis of the combined densitometry data presented in Supplementary Figure S2. Figure S4: Densitometry analysis of the Western blot data of p53. Figure S5: Statistical analysis of the combined densitometry data of L versus D melanocyte cultures in Supplementary Figure S4. Table S1. List of primary antibodies used in the Western blot experiments.
