# Supplementary material for: Differential Induction of Reactive Oxygen Species and Expression of Antioxidant Enzymes in Human Melanocytes Correlate with Melanin Content: Implications on the Response to Solar UV and Melanoma Susceptibility
Source: Antioxidants (Basel). 2022 Jun 20;11(6):1204. doi: 10.3390/antiox11061204 (PMC9219903; doi:10.3390/antiox11061204)
Supplement: Supplementary file 1 [file antioxidants-11-01204-s001.zip › antioxidants-1696974-supplementary.pdf]

Supplementary Figure S1. Solar UV-induced increase in ROS, expressed as percent of control of each of the cultures tested, based on the data presented in Fig. 3 b-e.

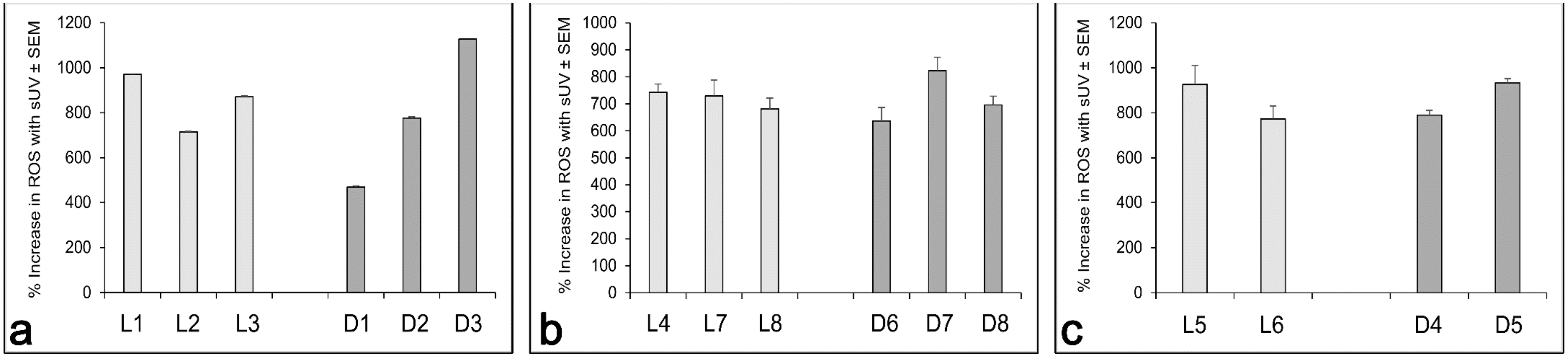

Supplementary Figures S2 a and b. Densitometry analysis of the Western blot data presented in Figure 4.

Supplementary Figure S2a Represents the densitometry of the Western blot in Fig. 4 a. Each band was normalized to its respective loading control (GAPDH or actin).

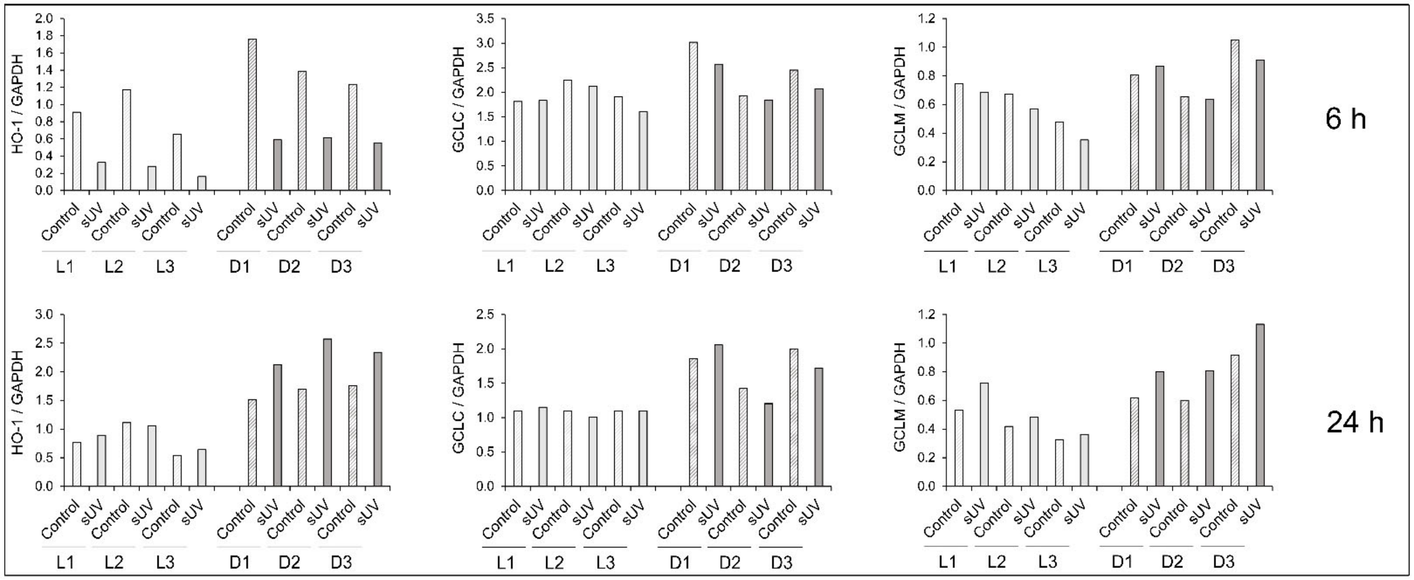

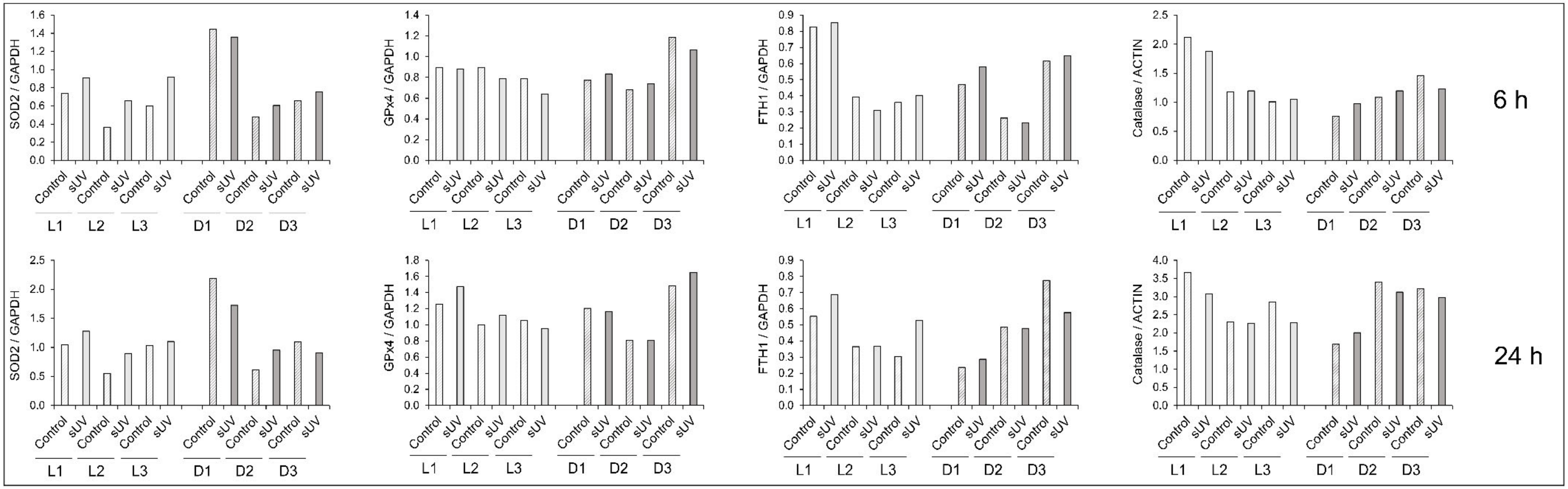

Supplementary Figure S2 a and b. Densitometry analysis of the Western blot data presented in Figure 4.

Supplementary Figure S2b Represents the densitometry of the Western blot in Fig. 4 b. Each band was normalized to its respective loading control (GAPDH or actin).

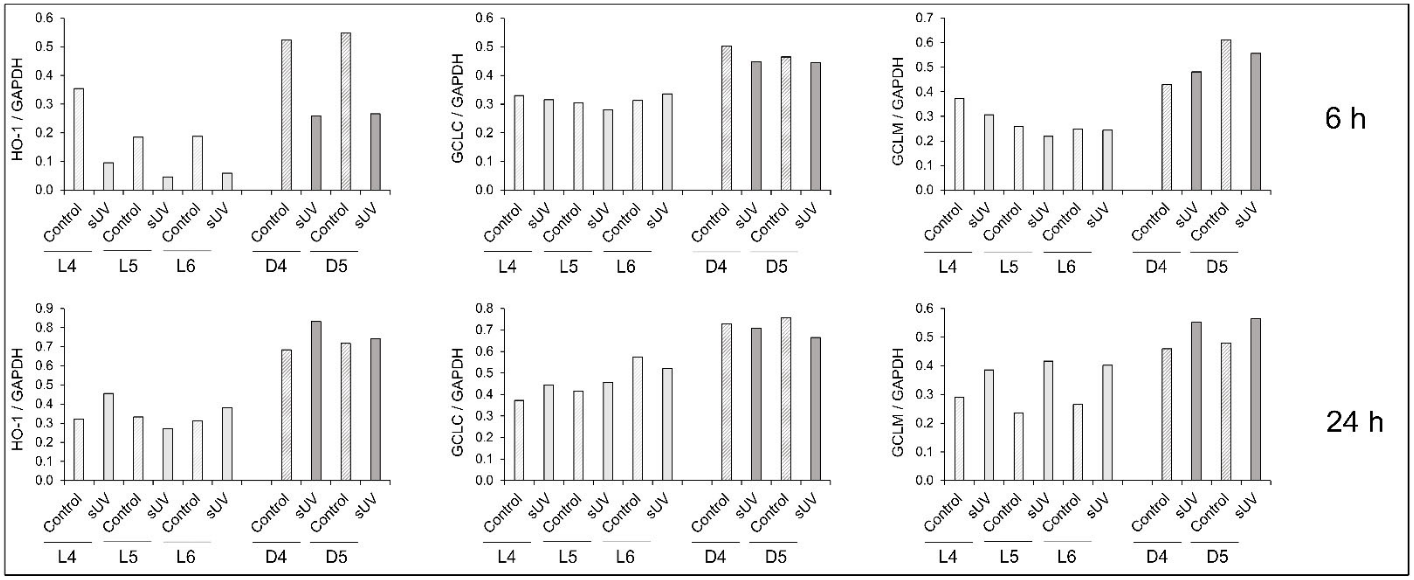

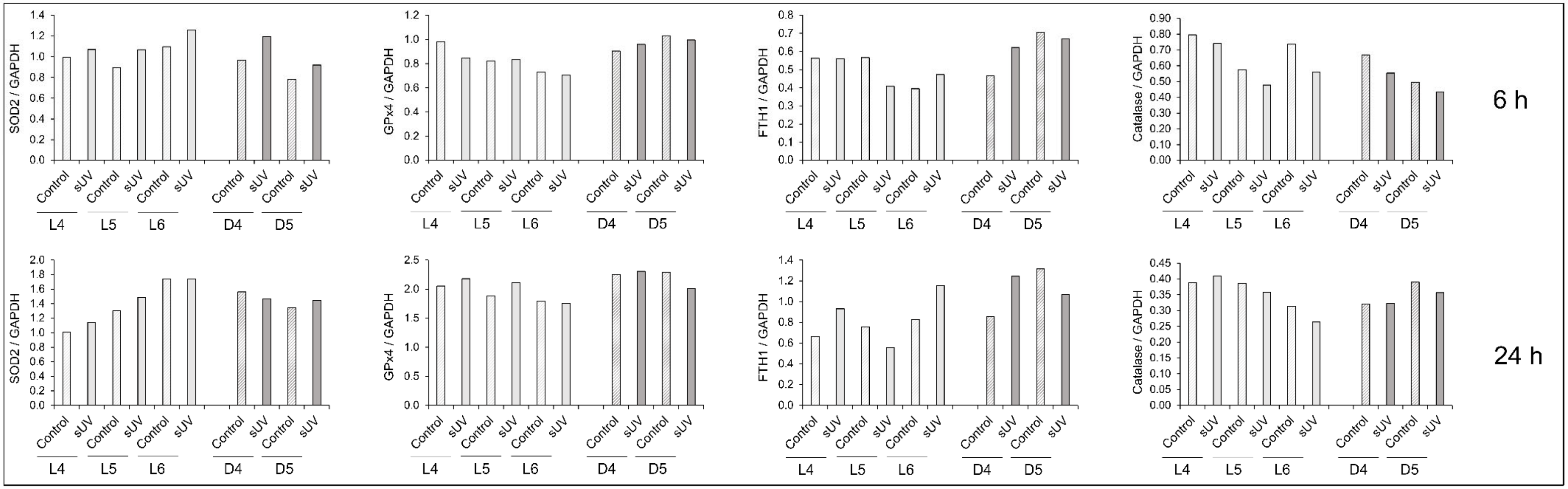

Supplementary Figure S3 a and b. Statistical analysis of the combined densitometry data presented in Supplementary Figure S2, representing basal levels of HO-1, GCLC, and GCLM in L versus D melanocyte cultures. Basal levels of HO-1 in (a) and (b) were statistically different in L as compared to D melanocytes at 6 h and 24 h time point. Basal levels of GCLC were statistically different in L versus D melanocytes at 6 h in (b), and at 24 h in (a) and (b). Basal levels of GCLM were statistically different in L versus D melanocytes at 6 h and 24 h in (b). *= P<0.05; **= P<0.01; ***= P<0.005; ****= P<0.001.

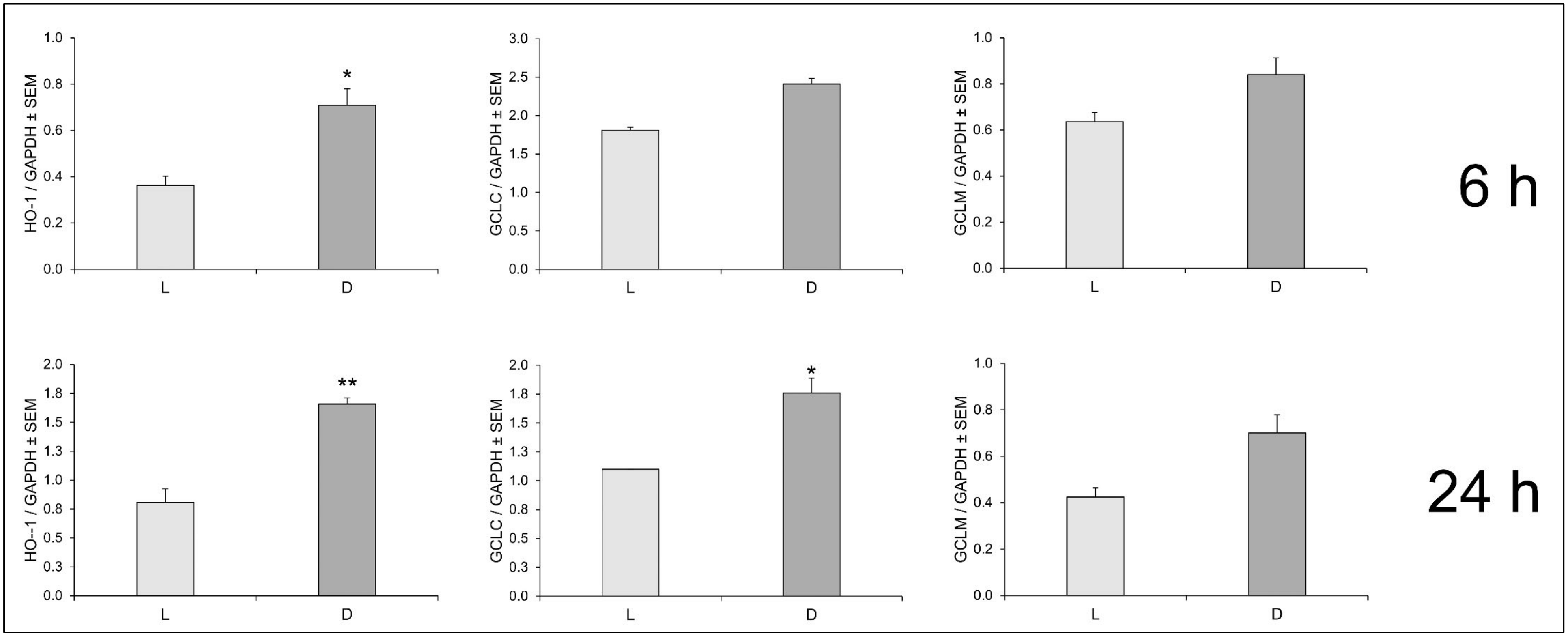

Supplementary Figure S3 a and b. Statistical analysis of the combined densitometry data presented in Supplementary Figure S2, representing basal levels of HO-1, GCLC, and GCLM in L versus D melanocyte cultures. Basal levels of HO-1 in (a) and (b) were statistically different in L as compared to D melanocytes at 6 h and 24 h time point. Basal levels of GCLC were statistically different in L versus D melanocytes at 6 h in (b), and at 24 h in (a) and (b). Basal levels of GCLM were statistically different in L versus D melanocytes at 6 h and 24 h in (b). *= P<0.05; **= P<0.01; ***= P<0.005; ****= P<0.001.

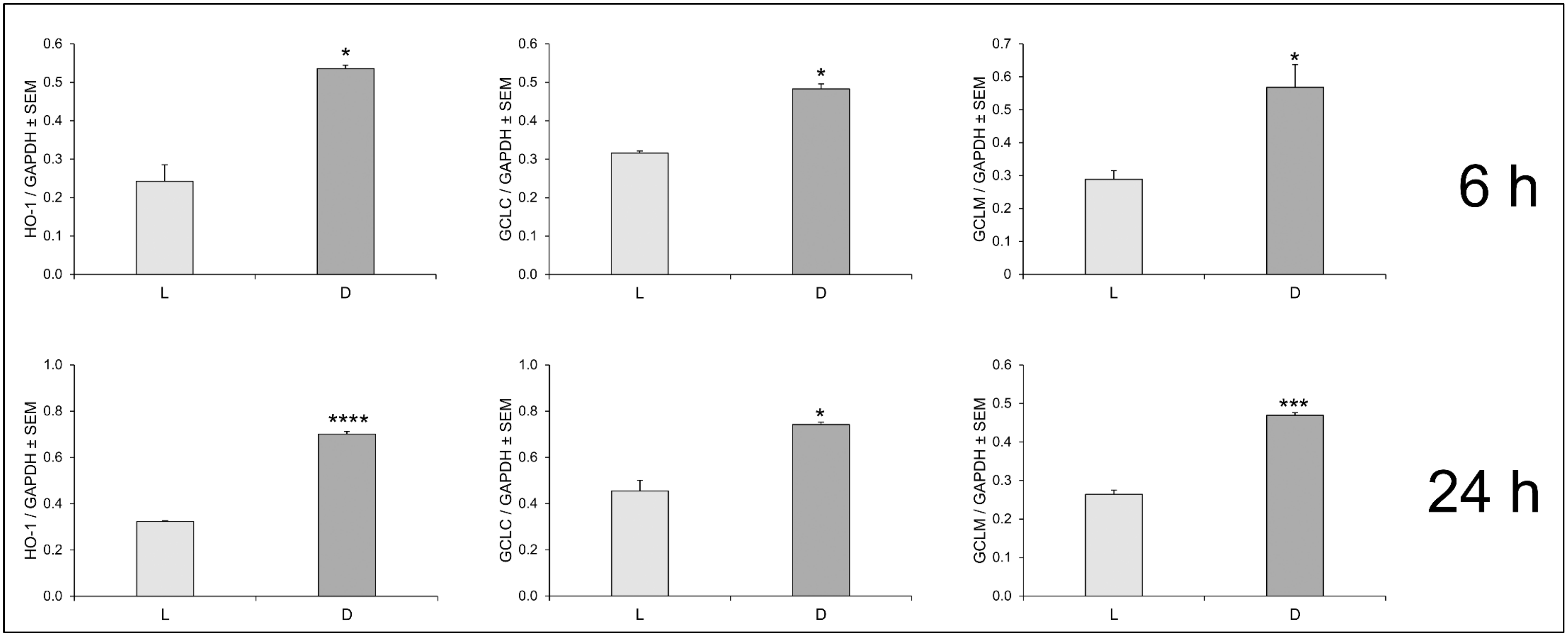

Supplementary Figure S4 a and b. Densitometry analysis of the Western blot data of p53, presented in Figure 6. **Supplementary Figure S4a** Represents the densitometry of the Western blot presented in Fig. 6 a.

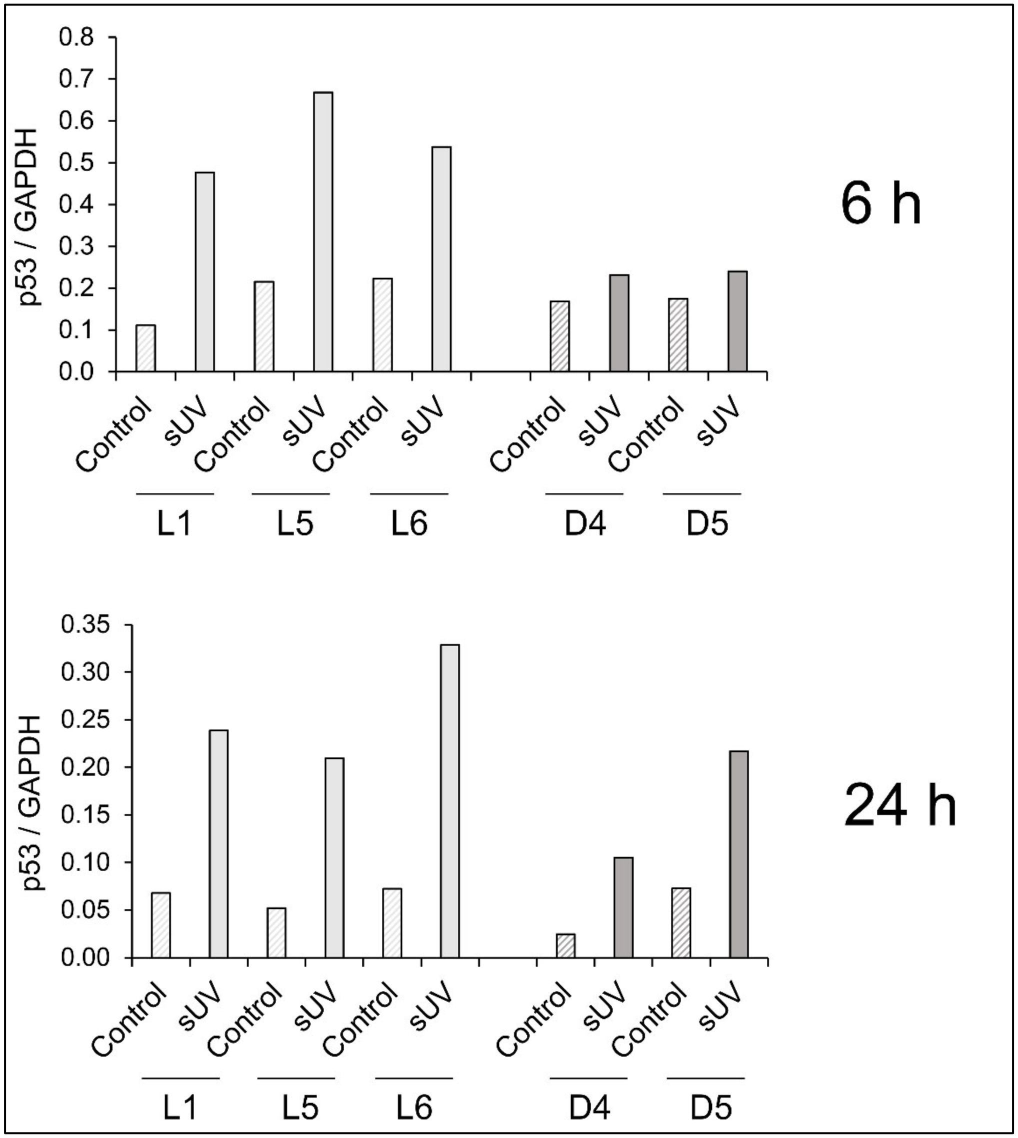

Supplementary Figure S4 a and b. Densitometry analysis of the Western blot data of p53, presented in Figure 6. **Supplementary Figure S4b** Represents the densitometry of the Western blot presented in Fig. 6 b, as compared to the respective GAPDH loading control.

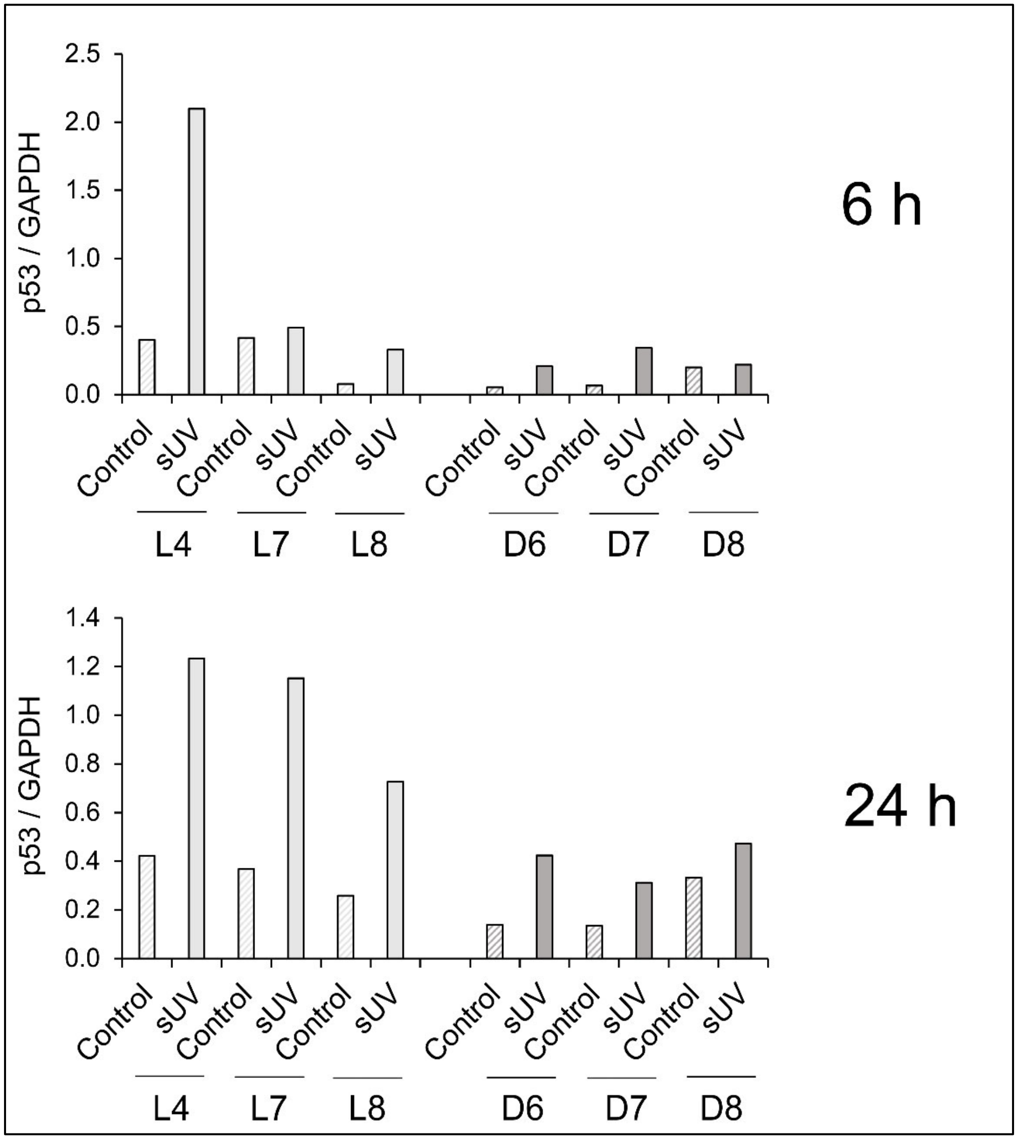

Supplementary Figure S5 a and b. Statistical analysis of the combined densitometry data of L versus D melanocyte cultures in Supplementary Figure S4.

Supplementary Figure S5a. Solar UV-induced p53 levels were significant different in L vs. D melanocytes at 6 h post solar UV in (a) (*= P<0.05).

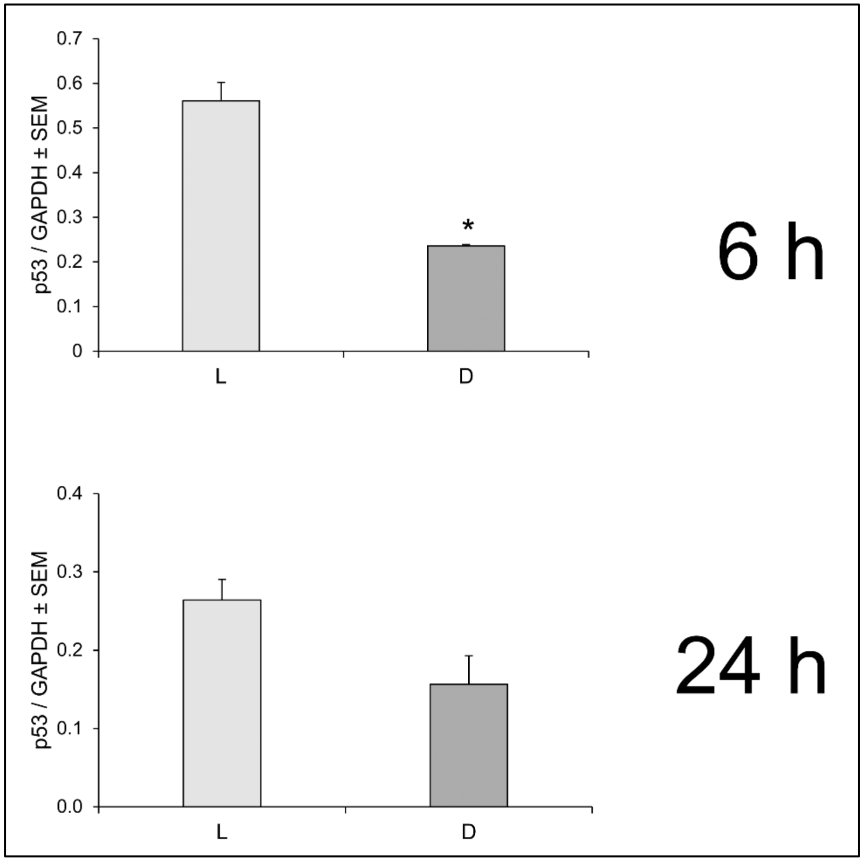

Supplementary Figure S5 a and b. Statistical analysis of the combined densitometry data of L versus D melanocyte cultures in Supplementary Figure S4.

Supplementary Figure S5b. Solar UV-induced p53 levels were significant different in L vs. D melanocytes at 24 h post UV in (b) (*= P<0.05).

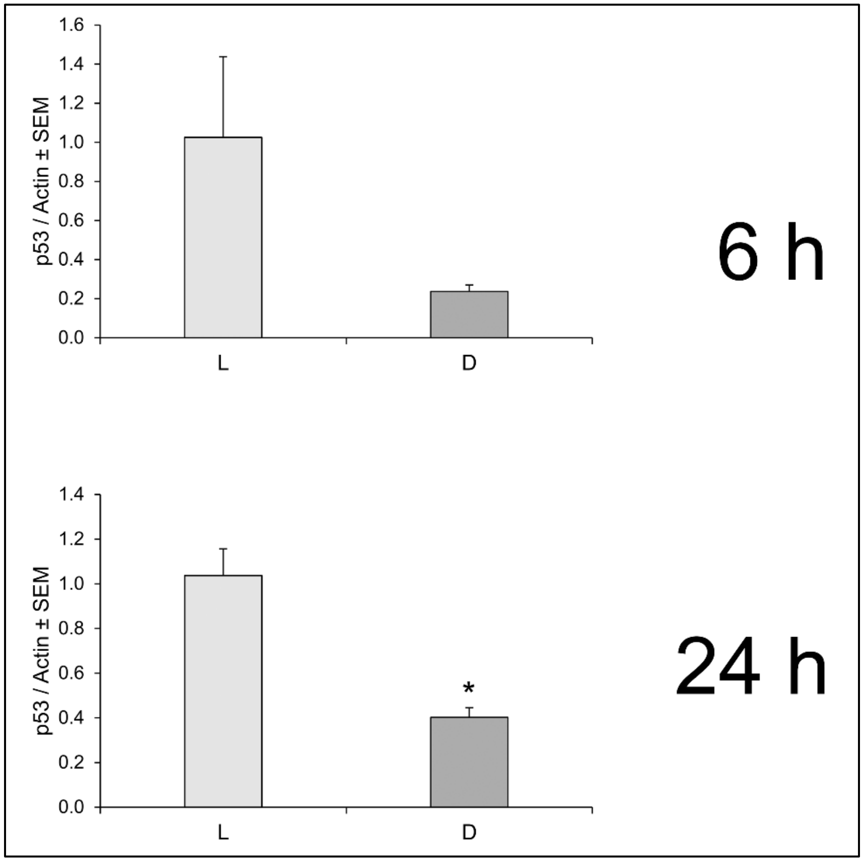

Supplementary Table S1. List of Primary antibodies used in Western blot experiments (Figures 4 and 6 a and b).

PROTEIN	SUPPLIER	CATALOG NUMBER	DILUTION	MOLECULAR WEIGHT (kDa)
**HO-1**	Cell Signaling Technology, Boston, MA, USA	70081	WB 1:1000	32
**GCLC**	Abcam, Cambridge, MA, USA	ab190685	WB 1:1000	73
**GCLM**	Proteintech, Rosemont, IL, USA	14241-1-AP	WB 1:1000	31
**SOD2**	Proteintech, Rosemont, IL, USA	24127-1-AP	WB 1:4000	25
**GPx4**	Abcam, Cambridge, MA, USA	ab125066	WB 1:5000	22
**FTH1**	Abcam, Cambridge, MA, USA	ab65080	WB 1:1000	21
**Catalase**	Abcam, Cambridge, MA, USA	ab209211	WB 1:2000	60
**p53**	Santa Cruz Biotechnology, Dallas, TX, USA	sc-126	WB 1:500	53
**Actin-HRP**	Santa Cruz Biotechnology, Dallas, TX, USA	sc-1615	WB 1:1000	43
**GAPDH-HRP**	Santa Cruz Biotechnology, Dallas, TX, USA	sc-47724	WB 1:1000	37
